# Predictors of Perceived Educational Relevance: A Regression Analysis Based on Teaching CanMEDS Roles

**DOI:** 10.1177/23821205251407752

**Published:** 2026-03-25

**Authors:** Verneri Hannula, Lari Lehtovirta, Petri Kulmala, Markku Sumanen

**Affiliations:** 1 7840 The Wellbeing Services County of Pirkanmaa, Tampere, Finland; 2 7840 Tampere University, Tampere, Finland; 3 60653 University of Oulu, Oulu, Finland; 4 Oulu University Hospital, Oulu, Finland

**Keywords:** CanMEDS, competency, preparedness, survey, undergraduate medical education

## Abstract

**Introduction:**

The CanMEDS framework, originally developed for postgraduate training, has also been adopted in undergraduate medical education. Evaluating how well undergraduate training prepares graduates for clinical work is one way to assess its success.

**Materials and methods:**

This study examined how Finnish doctors perceive the correspondence between their undergraduate education and their current work, and how the teaching of CanMEDS roles and other factors are associated with these evaluations. Data came from the Finnish nationwide Physician 2023 survey (response rate 49%). Of the 4882 respondents, 1240 had graduated between 2014 and 2023 and comprised the study group. Respondents assessed satisfaction with hospital and health centre training and with the teaching of the seven CanMEDS roles. Binary logistic regression was used to examine associations with perceived correspondence between undergraduate education and current work.

**Results:**

About 45% of respondents reported that their education corresponded well to their work. Nearly 80% were satisfied with hospital training, while only slightly more than half were satisfied with health centre training. The regression model explained 30% (Nagelkerke *R*^2^) of the variance in perceived correspondence, with a correct classification rate of 71%. Teaching of Medical knowledge (*OR* = 3.70, 95% CI [2.35–5.84]) showed the strongest association. Communication skills, Health Advocacy, and Leadership and Management skills were not significantly associated with perceived preparedness.

**Conclusions:**

Finnish doctors’ perceptions of undergraduate education aligning with work were strongly linked to the teaching of medical knowledge and lifelong learning. Hospital training contributed more to perceived preparedness than health centre training. Several CanMEDS roles showed limited association with preparedness.

## Introduction

The mission of undergraduate medical education is to prepare graduating students for their roles as doctors. However, the exponential growth of medical knowledge presents challenges for curricula to encompass all the essential information required for the diverse responsibilities of a doctor.^1^ It has been estimated that approximately 80% of a physician's competence is developed in the workplace.^2^

One way to determine the success of undergraduate medical education has been to evaluate graduates’ preparedness for work.^3^ There are wide range of studies assessing how medical school, gender or years since graduating affect doctors’ views of their preparedness to work after medical school.^3–5^ Studies have assessed general preparedness and preparedness for various work–life skills.^6^ In both cases the results of the studies have been mixed.^5,6^ Additionally, it has been stated that doctors’ perceptions about their undergraduate medical education should be further explored beyond the first year after their qualification.^3^

To help curricular development, various frameworks define learning outcomes for a graduating doctor. The CanMEDS framework has become the most widely accepted competency framework for medical doctors in the world.^7,8^ The framework outlines seven key roles essential for medical practice. These roles are interconnected, with ‘Medical Expert’ positioned at the centre of the CanMEDS diagram. The other roles include Professional, Communicator, Collaborator, Leader, Health Advocate, and Scholar.^7^ Although used commonly in postgraduate medical education, CanMEDS has also been adopted for undergraduate medical education.^7,9,10^ In Finland, there are nationwide competency goals for a graduating doctor established in 2020.^11^ Those competency goals are partly based on both CanMEDS and Tomorrow's Doctors’ frameworks.^7,12^

In addition to teaching medical expertise, Finnish medical schools provide education that encompasses all CanMEDS roles. For instance, communication skills are taught and assessed through simulated patient encounters with trained actors. Simulation-based teaching is also employed to strengthen clinical reasoning as well as leadership and collaboration competencies. Moreover, students record authentic patient consultations, which are subsequently reviewed and assessed together with clinical teachers. Despite the use of diverse educational methods, students frequently report a need for more structured feedback on communication skills, a greater number of simulation-based learning opportunities, and increased use of standardised patients.^13^

In Finland, all medical faculties conduct a portion of clinical teaching outside university hospitals, which has been recognised as a strength in national evaluations.^13^ Similarly, decentralisation is often perceived as beneficial for students.^14^ However, it has been suggested that a primary care perspective should be more consistently emphasised throughout medical education in Finland.^13^ Additionally, in the United Kingdom, it has been estimated that, on average, only 13% of clinical teaching takes place in primary care.^15^

Medical students’ appraisal of CanMEDS competencies have been studied.^9,16,17^ In a Dutch study, students rated the importance of different CanMEDS competencies and overall rated Professionalism the highest, followed by Communication.^16^ Similarly, in a Chinese study, Professionalism was rated second, while the Medical Expert role was rated the highest.^17^ Additionally, newly graduated doctors’ preparedness for CanMEDS skills has been assessed.^5^ Interns rated their own preparedness highest for the Collaborator role, followed by the Medical Expert role.^5^ Their supervisors, however, evaluated the interns’ preparedness lower in six out of the seven roles compared with the interns’ self-assessments, with the Professional role being the only exception.^5^ However, research on the implementation of CanMEDS competencies in undergraduate medical education remains limited. To assess the relevance of competency goals for graduating doctors, it is essential to evaluate how teaching these competencies influences preparedness for professional practice.

Therefore, we intended to study how undergraduate medical education corresponds to work–life demands in Finnish doctors who have graduated within 10 years. Our particular interest was to examine how doctors’ evaluations of teaching of seven CanMEDS roles and satisfaction with hospital and health centre training associate with their perception of how undergraduate education prepared them for work, as including these training settings helps contextualise CanMEDS teaching outcomes within overall clinical learning experiences.

## Materials and Methods

The Physician study 2023 is a part of nationwide surveys conducted every five years starting from 1988. The survey is conducted in collaboration with all five medical schools of Finland and the Finnish Medical Association.^18^

The questionnaire used in this study has not been formally validated. However, the long-term and consistent use of the same questions across survey waves supports the content validity and comparability of the measures. The questions in all the surveys have mostly been the same from the beginning of the series of surveys. However, since the 2018 survey, one question related to undergraduate medical education has been modified to follow the seven roles of the CanMEDS Framework.^7,19^

This study was conducted in Finland as a cross-sectional survey. The data were collected during the spring and summer of 2023 as part of the nationwide Physician Study 2023. The basic study population in the Physician 2023 study comprised all Finnish doctors under 70 years of age (*N* = 22 367). A random sample of 10 000 was drawn from the basic population. During the first part of data collection, the doctors in a random sample received an invitation to answer the questionnaire via email link. Later, the participants who had not yet answered received a postal paper questionnaire. The total number of respondents was 4882 (49%). For the present study, the inclusion criteria were doctors who had graduated between 2014 and 2023, as their experiences of undergraduate medical education were considered to best reflect the current situation. Exclusion criteria were graduation before 2014 or missing data on the year of graduation. In total, 1240 respondents met these criteria and comprised our study group.

We examined respondents’ demographics (gender, years since graduation, specialisation phase). Additionally, we assessed four questions addressing respondents’ undergraduate medical education. Those questions and their answer options were following:

1. How does the undergraduate medical education you received correspond to your work? (Very poorly, fairly poorly, moderately, fairly well, very well)
2. How satisfied are you with the hospital work training of your undergraduate medical education?
3. How satisfied are you with the health centre training of your undergraduate medical education?
  (Very dissatisfied, fairly dissatisfied, hard to say, fairly satisfied, very satisfied)

And the question addressing the seven roles of the CanMEDS Framework:

4. To what extent did you receive teaching and guidance on the following aspects during your undergraduate medical education?
  - Medical knowledge
  - Learning strategies
  - Communication skills
  - Collaboration skills
  - Professionalism
  - Leadership and management skills
  - Health advocacy
    (Far too little, too little, just right, too much, far too much)

This study conforms to the STROBE statement for observational studies.^20^

### Statistical analysis

A binary logistic regression model was structured to examine how the following aspects are related to the assessment of how the undergraduate medical education received corresponds to work.

1. Gender
2. Years since graduation
3. Assessments of teaching of different work–life skills
4. Assessments of satisfaction for hospital work and health centre training

The question of how the undergraduate medical teaching received corresponds to work was categorised into two classes regarding the answer options. First class contained very poorly, fairly poorly and moderately (reference category), and second class contained fairly well and very well. Answer option moderately was categorised with options poorly, because we wanted to examine what factors are particularly related to assessments of upright correspondence of undergraduate medical education and respondents work. In the regression model, the response options ‘too much’ and ‘far too much’ were treated as missing values in questions addressing the seven roles of the CanMEDS Framework.

The forward stepwise binary logistic regression model was used to select the final significant independent variables for the model. All candidate variables, including all seven work–life skills, satisfaction with clinical training, gender and years since graduation were initially entered simultaneously. The stepwise procedure then retained only those variables that were statistically significant in predicting the outcome, resulting in a final model including four work–life skills (Medical knowledge, Collaboration skills, Learning strategies, and Professionalism), satisfaction with hospital and health centre training, gender, and years since graduation.

We conducted the analysis using SPSS Version 29, considering results significant if *p* < 0.05. Additionally, we calculated odds ratios with 95% confidence intervals.

## Ethics

The study did not involve any patient data, and the responding was not mandatory. Respondents were informed about the use of the questionnaires; hence, it can be stated that they gave written informed consent when they answered. The research followed the World Medical Association Declaration of Helsinki and the Finnish National Advisory Board on Research Integrity guidelines.^21,22^ Consequently, an ethical statement was not required according to Finnish national regulations.^22,23^

## Results

The demographic information of the respondents’ is presented in Table 1. Almost 60% of the respondents were woman and roughly half were specialising doctors. Around 45% of the respondents assessed that the undergraduate medical education received corresponds to their work fairly or very well (Table 2). Almost 80% of the respondents were satisfied with the hospital work training received, but respectively only slightly over half were satisfied with health centre training.

**Table 1.** Demographic Information of the Respondents (N = 1240).

Variable	Categories	*n*	%
Gender	Woman	723	58.3
	Male	506	40.8
	Other/missing	11	0.9
Years since graduation	0–1	236	19.1
	2–3	315	25.4
	4–5	267	21.5
	6–7	202	16.3
	8–9	220	17.7
Specialising phase	Not specialised	501	40.4
	Specialising	608	49.0
	Specialised	131	10.6

**Table 2.** Respondents' Evaluations of How Medical Education Corresponds to Their Work (*n* = 1237) and Satisfaction with Hospital (*n* = 1236) and Health Centre (*n* = 1237) Training.

Question	Very Poorly	Fairly Poorly	Moderately	Fairly Well	Very Well
How does the undergraduate medical education you received correspond to your work?	22 (1.8)	189 (15.3)	476 (38.5)	484 (39.0)	66 (5.3)
How satisfied are you with the following aspects of your undergraduate medical education:	Very dissatisfied	Fairly dissatisfied	Hard to say	Fairly satisfied	Very satisfied
Hospital work training	9 (0.7)	123 (10.0)	134 (10.8)	856 (69.3)	114 (9.2)
Health centre training	54 (4.4)	329 (26.6)	196 (15.8)	576 (46.6)	82 (6.6)

Percentages in parentheses.

**Figure 1.**
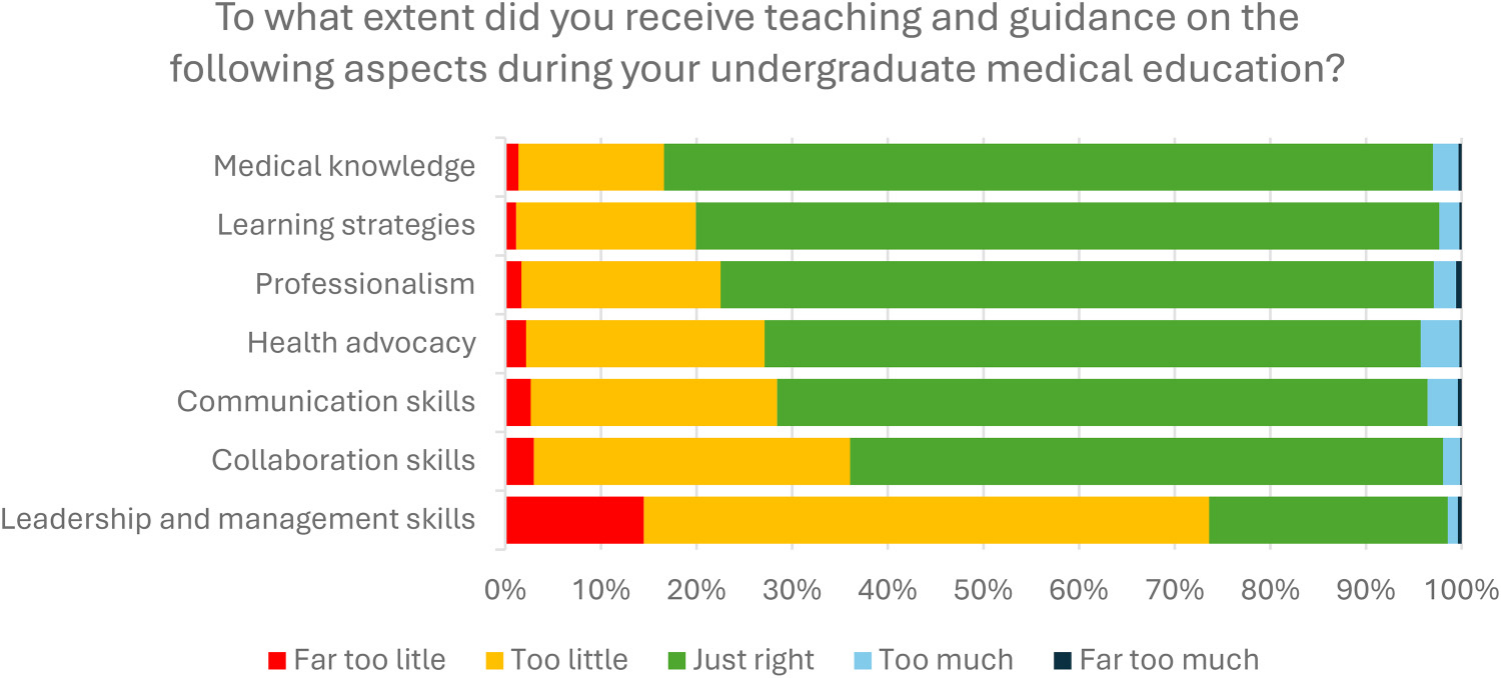
Respondents’ evaluations of the teaching of various work–life skills during undergraduate medical education.

Evaluations regarding teaching and guidance of different work–life skills are presented in Figure 1. Overall, respondents were generally quite satisfied with the teaching, as over 60% reported receiving an appropriate amount of instruction and guidance in six out of the seven work–life skills. Medical knowledge received the best evaluation and 80% of the respondents assessed they had received just right amount of teaching and guidance regarding it. Leadership and management skills received clearly the worst evaluations and 75% of the respondents thought that they had received too little teaching and guidance on that aspect.

Table 3 presents the results of the binary logistic regression analysis examining factors associated with how well respondents felt their undergraduate medical education prepared them for work. The model explained 30% of the variance in perceived correspondence (Nagelkerke *R*^2^ = 0.295) and correctly classified 71% of cases.

**Table 3.** Binary Logistic Regression Model.

Question	Predictor	B	*OR*	95% Confidence Interval
To what extent did you receive teaching and guidance on the following aspects during undergraduate medical education				
	Medical knowledge	1.31	3.70	[2.35–5.84]
	Learning strategies	0.69	2.00	[1.38–2.89]
	Collaboration skills	0.66	1.93	[1.45–2.57]
	Professionalism	0.42	1.52	[1.08–2.14]
How satisfied are you with the following aspects of your undergraduate medical education				
	Hospital work training	0.60	1.81	[1.45–2.28]
	Health centre training	0.39	1.48	[1.28–1.72]
Gender	Female	−0.29	0.75	[0.56–0.99]
Years since graduation	Per year	−0.66	0.94	[0.89–0.98]

Dependent variable: ‘How does the undergraduate medical education you received correspond to your work?’ Response options for the dependent variable are categorised into two classes: 0 = poorly and moderately (reference category), and 1 = well. The variables included in the analysis but excluded from the final model (*p* > 0.05) were assessments of teaching in: (1) communication skills, (2) health advocacy, and (3) leadership and management skills. Nagelkerke *R*^2^ = 29.5%. *B* = log-odds coefficient, *OR* = odds ratio. (*n* = 1045).

Four work–life skills-related variables—Medical knowledge (*OR* = 3.70), Learning strategies (*OR* = 2.00), Collaboration skills (*OR* = 1.93), and Professionalism (*OR* = 1.52)—were significantly associated with higher perceived correspondence. This indicates that respondents who rated these aspects of teaching highly were more likely to report that their education prepared them well for work. Satisfaction with hospital work training (*OR* = 1.81) and health centre training (*OR* = 1.48) also contributed positively to perceived preparedness. Gender and years since graduation had smaller but significant effects, with males and more recent graduates slightly more likely to report high correspondence.

Three work–life skills—Communication skills, Health advocacy, and Leadership and management skills—were included in the initial analysis but were not retained in the final model, suggesting they did not independently predict perceived preparedness when other factors were accounted for. Overall, the results highlight the particular importance of core work–life skills and clinical training experiences in shaping graduates’ perceptions of how well their education prepared them for practice.

## Discussion

Slightly less than half of the respondents reported that their undergraduate medical education corresponded well with their current work. While respondents were generally satisfied with their hospital training, evaluations of health centre training were lower.

The teaching of Medical knowledge had the greatest impact on respondents’ evaluations of how well undergraduate medical education corresponded to their current job. This is logical, as medical knowledge is central to a doctor's work. Similarly, the CanMEDS framework places ‘Medical Expert’ at its core. Previous research from China has also shown that students considered the Medical Expert role to be the most important role.^17^ Similarly, German supervising clinicians rated training Medical Expert competencies as the most important for final-year students.^24^ However, in a study of Dutch students, the Medical Expert role was rated only the third most important role, following Professionalism and Communicator.^16^ In our study, medical knowledge received the highest evaluations among different work–life skills for its teaching and guidance. In a previous study, interns and their supervisors assessed that newly graduated doctors were best prepared for the Collaborator role, with the Medical Expert role ranking second.^5^

A rapidly growing scientific knowledge base is a challenge in medical education.^1^ Hence, lifelong learning is an essential skill for every doctor.^25^ Therefore, it is not surprising that teaching of Learning strategies associated with how undergraduate education corresponds to work.

Similarly, Collaboration skills and Professionalism were included as independent variables in our regression model. German clinicians rated the Collaborator role as the most important for their own daily work.^24^ In contrast, Ringsted et al found that the Collaborator role received the second-lowest ratings in importance among the CanMEDS roles, ranking just above Health Advocate.^8^ The importance of the Professional role has generally been rated highly in studies,^8,17,26^ and final-year Dutch students ranked it as the most important role.^16^ It can be assumed that both collaboration skills and professionalism are essential regardless of a doctor's specific role, emphasising the importance of teaching them in undergraduate medical education.

Slightly surprisingly, evaluations of Communication skills teaching did not significantly associate with perceptions of how well undergraduate education corresponded to work. The importance of the Communicator role was highly ranked by doctors across all specialities in a Danish study.^8^ Likewise, Jilg et al found that supervising clinicians rated the Communicator role as the second most important CanMEDS role to teach (after Medical Expert) and the second most important in their own work (after Collaborator).^24^ Similarly, the Communicator role has been highly ranked in other studies by both students and faculty.^16,17^ Watmough et al conducted interviews with medical graduates and found that, despite the absence of specific communication classes in their education, graduates felt they were competent communicators, having acquired these skills naturally or through observing doctors.^27^ Additionally, physicians may perceive that they develop communication skills through the course of their clinical practice. This may help explain our finding that the teaching of Communication skills was excluded from the regression model.

Likewise, Leadership and management skills had no significant association with the dependant variable in the regression model. Additionally, those skills received the lowest evaluations for their teaching among various work–life skills. This aligns with a German study, which found that the competencies associated with the Leader/Manager role were not well integrated into the curricula of eight medical schools studied.^28^ The importance of the Manager role has generally been rated lower than several other CanMEDS roles.^24,26^ Additionally, in Finland, mandatory leadership training is required for all doctors during their specialisation phase. This may help explain our finding that the teaching of leadership and management skills in undergraduate education does not correlate with how well education corresponds to work.

The third CanMEDS role that was excluded from the regression model was the evaluation of Health Advocacy teaching. Our earlier study with the same data, which addressed doctors working in a health centre, revealed that Health advocacy teaching was one of the variables associated with education correspondence with work in that subgroup.^29^ Similarly, Ringsted et al found that all specialities, except those in general practice and social medicine, rated the Health Advocate role as the least important.^8^ In contrast, these two specialities ranked it as the second most important role.^8^ It might be assumed that doctors think health advocacy is mostly important in primary health care. Additionally, in previous studies medical students have ranked Health advocate among the least important CanMEDS roles.^16,17^

Goldacre et al compared doctors who had graduated one year earlier with those who had graduated three years earlier and found that the three-year graduates were more critical of how well medical school had prepared them for work. ^30^ That aligns with our finding that the years since graduating negatively affected the perception of how undergraduate education corresponds to work.^3^ Miles et al reported that gender influenced only one subscale of preparedness (dialogue with patients) with results favouring females.^4^ Nevertheless, the observed gender differences across studies have generally been modest.^6^

The regression model explained only 30% of the variance in the dependent variable, indicating that several unidentified factors may influence how doctors assess the correspondence between their undergraduate medical education and their job. These factors may include, for example, the respondents’ speciality, work environment and personal characteristics. This study did not include a priori sample size calculation, as it was based on an existing data set of all available respondents. Therefore, the statistical power was determined by the size of the existing sample rather than by an a priori design consideration. The questionnaire has not undergone a formal validation process. Nevertheless, it has been used consistently in national surveys over several decades, which supports the reliability and comparability of the measures.

We focused only on the seven roles and did not examine their key concepts or sub-competencies, which can be considered a limitation of the study. Additionally, the wording of the questions and roles differed slightly from the original CanMEDS framework, making comparisons with other studies somewhat imprecise. For example, our study included ‘learning strategies’ instead of the Scholar role. Furthermore, we did not compare responses from doctors across different specialities, which could have provided additional insights.^8^ However, undergraduate medical education cannot be tailored solely to the needs of specific specialities. Hence, evaluating the perceptions of a broad range of doctors from different specialities can be considered a strength of this study. Additionally, our study included doctors at various career stages, and the respondents’ graduation years varied, providing a more comprehensive perspective.

Previous studies have shown that the importance of all CanMEDS roles has generally been rated quite high, with no consistent statistically significant differences between them.^8,16,17,26^ Additionally, the amount of teaching related to different roles varies.^9^ This study provides new insight into how teaching these roles affects perceived preparedness for work. The survey was conducted nationwide, and a response rate of nearly 50% can be considered reasonable.

## Conclusions

Our study supports the centrality of the Medical Expert role in the CanMEDS framework, as perceived by graduated doctors. Teaching medical knowledge is a fundamental aspect of undergraduate medical education, and it had the greatest impact on how doctors perceived their education corresponded to their work. However, the teaching of several, though not all, of the other CanMEDS roles also influenced these perceptions. Our findings highlight the value of aligning undergraduate curricula with the CanMEDS framework by ensuring sufficient emphasis on core professional, collaborative, and medical knowledge skills. Practically, this could involve designing clinical rotations and teaching sessions that clearly connect these roles to real-world practice.

Future research could explore whether doctors perceive differences in which CanMEDS roles should be emphasised in undergraduate education versus postgraduate training. In particular, the finding that the teaching of communication skills was excluded from the regression model is surprising and could require further investigation.

## Supplemental Material

sj-docx-1-mde-10.1177_23821205251407752 - Supplemental material for Predictors of Perceived Educational Relevance: A Regression Analysis Based on Teaching CanMEDS Roles Supplemental material, sj-docx-1-mde-10.1177_23821205251407752 for Predictors of Perceived Educational Relevance: A Regression Analysis Based on Teaching CanMEDS Roles by Verneri Hannula, Lari Lehtovirta, Petri Kulmala and Markku Sumanen in Journal of Medical Education and Curricular Development

sj-docx-2-mde-10.1177_23821205251407752 - Supplemental material for Predictors of Perceived Educational Relevance: A Regression Analysis Based on Teaching CanMEDS Roles Supplemental material, sj-docx-2-mde-10.1177_23821205251407752 for Predictors of Perceived Educational Relevance: A Regression Analysis Based on Teaching CanMEDS Roles by Verneri Hannula, Lari Lehtovirta, Petri Kulmala and Markku Sumanen in Journal of Medical Education and Curricular Development
