# Supplementary material for: Predictors of Perceived Educational Relevance: A Regression Analysis Based on Teaching CanMEDS Roles
Source: J Med Educ Curric Dev. 2026 Mar 25;13:23821205251407752. doi: 10.1177/23821205251407752 (PMC13018681; doi:10.1177/23821205251407752)
Supplement: sj-docx-1-mde-10.1177_23821205251407752 - Supplemental material for Predictors of Perceived Educational Relevance: A Regression Analysis Based on Teaching CanMEDS Roles [file sj-docx-1-mde-10.1177_23821205251407752.docx]

**Predictors of Perceived Educational Relevance: A Regression Analysis Based on teaching CanMEDS roles**

The questionnaire used in the survey. Translated from the original Finnish questionnaire.

1. Gender

Male, female, other, I don’t want to report

1. In which year did you graduate as a physician?

__________________________

1. Status of specialization training

Not specialized, specializing, specialized

1. How does the undergraduate medical education you received correspond to your work? Very poorly, fairly poorly, moderately, fairly well, very well
2. How satisfied are you with the hospital work training of your undergraduate medical education?

Very dissatisfied, fairly dissatisfied, hard to say, fairly satisfied, very satisfied

1. How satisfied are you with the health centre training of your undergraduate medical education?

Very dissatisfied, fairly dissatisfied, hard to say, fairly satisfied, very satisfied

1. To what extent did you receive teaching and guidance on the following aspects during your undergraduate medical education?

|  | far too little | too little | just right | too much | far too much |
| --- | --- | --- | --- | --- | --- |
| - Medical knowledge |  |  |  |  |  |
| - Learning strategies |  |  |  |  |  |
| - Communication skills |  |  |  |  |  |
| - Collaboration skills |  |  |  |  |  |
| - Professionalism |  |  |  |  |  |
| - Leadership and management skills |  |  |  |  |  |
| - Health advocacy |  |  |  |  |  |
